# Longitudinal relationship of liver injury with inflammation biomarkers in COVID-19 hospitalized patients using a joint modeling approach

**DOI:** 10.1038/s41598-022-09290-x

**Published:** 2022-04-01

**Authors:** Carla Diaz-Louzao, Lucia Barrera-Lopez, Maria Lopez-Rodriguez, Clara Casar, Nestor Vazquez-Agra, Hadrian Pernas-Pardavila, Ana Marques-Afonso, Martin Vidal-Vazquez, Jonathan G. Montoya, Ariadna H. Andrade, Ivan Fernandez-Castro, Pablo Varela, Arturo Gonzalez-Quintela, Esteban Otero, Francisco Gude, Carmen Cadarso-Suarez, Santiago Tome

**Affiliations:** 1 Department of Psychiatry, Radiology, Public Health, Nursing and Medicine, University of Santiago de Compostela, Santiago de Compostela, Spain; 2 Epidemiology and Research Unit, Hospital Clinico Universitario de Santiago, Instituto de Investigaciones Sanitarias (IDIS) Santiago de Compostela, Santiago de Compostela, Spain; 3 Hepatology Unit, Department of Internal Medicine, Hospital Clínico Universitario de Santiago de Compostela, Travesia da Choupana S/N, 15705 Santiago de Compostela, Spain; 4 CITMAga, Santiago de Compostela, Spain; 5 Department of Statistics, Mathematical Analysis and Optimizacation, Universitiy of Santiago de Compostela, Santiago de Compostela, Spain

**Keywords:** Hepatology, Viral infection

## Abstract

The mechanisms underlying liver disease in patients with COVID-19 are not entirely known. The aim is to investigate, by means of novel statistical techniques, the changes over time in the relationship between inflammation markers and liver damage markers in relation to survival in COVID-19. The study included 221 consecutive patients admitted to the hospital during the first COVID-19 wave in Spain. Generalized additive mixed models were used to investigate the influence of time and inflammation markers on liver damage markers in relation to survival. Joint modeling regression was used to evaluate the temporal correlations between inflammation markers (serum C-reactive protein [CRP], interleukin-6, plasma D-dimer, and blood lymphocyte count) and liver damage markers, after adjusting for age, sex, and therapy. The patients who died showed a significant elevation in serum aspartate transaminase (AST) and alkaline phosphatase levels over time. Conversely, a decrease in serum AST levels was observed in the survivors, who showed a negative correlation between inflammation markers and liver damage markers (CRP with serum AST, alanine transaminase [ALT], and gamma-glutamyl transferase [GGT]; and D-dimer with AST and ALT) after a week of hospitalization. Conversely, most correlations were positive in the patients who died, except lymphocyte count, which was negatively correlated with AST, GGT, and alkaline phosphatase. These correlations were attenuated with age. The patients who died during COVID-19 infection displayed a significant elevation of liver damage markers, which is correlated with inflammation markers over time. These results are consistent with the role of systemic inflammation in liver damage during COVID-19.

## Introduction

Since the notification at the end of December 2019 of several cases of pneumonia caused by severe acute respiratory syndrome coronavirus 2 (SARS-CoV-2), the infection has spread rapidly throughout the world, reaching pandemic levels. As of the beginning of June 2021, the pandemic has caused over 173 million infections and more than 3.5 million deaths. The disease caused by this agent has been coined coronavirus disease 2019 (COVID-19) by the World Health Organization^1^. The main organ affected is the lungs, and most patients present respiratory symptoms; however, gastrointestinal symptoms and especially liver damage are also frequent during the infection^2–4^.

The presence of liver damage during the course of SARS-CoV-2 infection has been reported in patients with and without previous liver disease^5–8^. This involvement is characterized by a predominant elevation of alanine transaminase (ALT) and aspartate transaminase (AST) levels, which affects 15–62% of patients and can be accompanied by significant increases in bilirubin levels. Most of these alterations are characterized by being mild-moderate transient increases and are related to the severity of the viral infection. Autopsies of some patients who died during infection have shown liver abnormalities compatible with hepatic steatosis and portal inflammation^9^.

The exact mechanism of liver damage is currently unknown, but various hypotheses have been proposed. There are at least 3 types of receptors for the virus in the liver (angiotensin-converting enzyme 2, transmembrane serine protease 2, and furin), distributed in cholangiocytes and hepatocytes, which suggests that the direct action of the virus on liver cells induces liver damage, in a similar manner as at the pulmonary level, by means of a virus-receptor interaction^9,10^. There is also the possibility that the liver damage is related to the degree of systemic inflammatory response, in the scenario of the so-called “cytokine storm” and that the liver behaves as an innocent bystander. Anoxia, which is secondary to the respiratory involvement that occurs mainly in the most severe cases of infection, could lead to sustained hypoxia of the liver tissue, with liver cell damage consequently. Lastly, the drug toxicity derived from the multiple treatments administered, especially during the first wave, could explain the subsequent liver damage due to hepatotoxicity^11^.

An exhaustive review of articles published to date generally shows a “still photo” of liver disorders in COVID-19, such as altered transaminase and bilirubin levels at a certain point in the disease progression. Few studies have dynamically analyzed these alterations over time, and even fewer have studied their potential relationships with biological markers such as inflammation markers (IMs)^3,12–16^. The moment a liver disorder appears can indicate whether there is any relationship with the inflammatory response, drug administration, or tissue anoxia and can infer whether the damage is due to the direct action of the virus. Specifically directed studies, with repeatedly and exhaustively collected variables, might therefore shed light on whether the temporary establishment of liver disorders is due to the immediate direct effect of the virus or to other mechanisms. The intention of this study is to describe the liver disorders in a cohort of patients from our institution corresponding to the “first wave” of COVID-19. By employing an innovative statistical joint modeling methodology, we seek to investigate the dynamics of the modifications in transaminase levels analyzed longitudinally over time and to correlate them with IMs, C-reactive protein (CRP), interleukin-6 (IL-6), D-dimer, and lymphocytes as a function of survival.

## Patients and methods

Data were collected from the medical reports of patients diagnosed with COVID-19 and admitted to the Complejo Hospitalario Universitario of Santiago de Compostela in Spain, from March 12, 2020 (date of first COVID-19 diagnosis) to April 11, 2020. A confirmed case of COVID-19 was defined as a positive result in the reverse transcription polymerase chain reaction (RT-PCR) test on samples obtained from nasal or throat swabs performed in accordance with WHO protocol^17^. The study time for each included patient was from admission until the patient died or was admitted to the intensive care unit.

Using Python 3.8, a specific program with a graphical user interface was designed through which each patient’s clinical data were imported and automatically recorded from the electronic medical record. In all cases, the data were encrypted prior to filing. We collected all demographic data, admission history, and medical comments on the patient's progression, clinical constants (heart rate, blood pressure, and body temperature), pulse oximetry, radiological studies, and pharmacological prescriptions, including the date and time of their prescription. Among the laboratory determinations, we recorded all the parameters of the complete blood count, and blood biochemistry (glucose, urea, creatinine, uric acid, sodium, potassium, calcium, magnesium, albumin, cholesterol, triglycerides, AST, ALT, gamma glutamyl aminotransferase [GGT], Alkaline phosphatase [ALP], bilirubin, and coagulation), which included the international normalized ratio, prothrombin, blood gases (pO_2_, pCO_2_, O_2_ saturation, bicarbonate, lactate), procalcitonin, lactate dehydrogenase, vitamin D, and hemoglobin A1c. We considered CRP, D-dimer, IL-6, and lymphocyte counts as a specific group of IMs and recorded the reference limits of normality for each parameter and laboratory measurement. An abnormal increase in transaminase levels has been considered any value that exceeds the normal range. The transaminase elevation profile has been grouped according to R-factor and similar to how drug-induced liver damage (DILI) is classified depending on whether the predominant elevation is due to AST and ALT (hepatocellular pattern), GGT and ALP(cholestatic pattern) or elevation similar to one another (mixed).The *R*-value is defined as serum alanine aminotransferase/upper limit of normal (ULN) divided by serum alkaline phosphatase/ULN. By common convention, *R* ≥ 5 is labeled as hepatocellular, *R* < 2 is labeled as cholestatic, and 2 < *R* < 5 is labeled as “mixed”^18^.

For those cases in which specific treatment had been started before hospital admission, the start date of each drug was manually recorded. The program classified each drug into its corresponding pharmacological group, adding this as an additional variable in each registry. The records were processed and archived in a relational sqlite3 database, with independent tables for physical constants, pharmacological prescriptions, symptoms, exploratory signs, radiological signs, laboratory parameters, complications, and status (emergency room, hospital ward, critical unit, discharge or death), indexed by case number and start date and linked to a general table with demographic characteristics, antecedents, and form of infection.

### Ethical issues

The study was conducted in accordance with the guidelines of the Declaration of Helsinki and the principles of good practice and was approved by the Institutional Review Board of the Galician health service on April 3, 2020 (# 2020/194). Informed consent documents were waived by the Institutional Review Board.

### Statistical analysis

A descriptive analysis of the sample was performed according to the demographic variables, treatments administered, and absolute values of transaminases and IMs for the total sample and separately for the patients who were eventually discharged or died. The continuous variables are expressed as median and interquartile range, and the categorical variables are expressed as their absolute frequency and percentage. Differences between the surviving and deceased patients were assessed by means of Fisher’s exact test for binary variables; the Mann–Whitney *U* test was employed for the continuous variables.

Given that not all blood samples were analyzed with the same apparatus, the raw values of certain variables were not comparable. Therefore, prior to any statistical analysis, we divided the raw values for transaminases, IL-6, and CRP by their maximum normal values a form of weighting. For these variables, we thereby obtain the proportion with respect to the acceptable maximum (< 1 implies normal levels, and > 1 implies high levels). At this point, given that transaminases and IMs do not follow a Gaussian distribution, we estimated the exploratory curves for these variables over time for the deceased and living patients by the least absolute shrinkage and selection operator regression analysis^19^, given that this technique does not assume any parametric distribution for data.

We then normalized the transaminases, D-dimer, and IL-6 values using a Box–Cox transformation to facilitate statistical modeling calculations. We considered that transforming the CRP and lymphocytes values would not produce any improvement, so we decided to use the raw data for these covariates.

To assess the factors that could influence the transaminase values, we fitted a generalized additive mixed model (GAMM)^20^ for each Box–Cox-transformed transaminase, adjusting for sex, exitus (binary for alive or deceased), antiviral treatment (binary for lopinavir/ritonavir and/or hydroxychloroquine administration), age, IM-exitus association, and time-exitus association. We also included random intercepts by patient to consider the variability within each individual (the values recorded for the same individual are expected to be more closely related to each other than to the values of another individual). The smooth effects of continuous covariates were considered by means of thin-plate spline smoothers. The GAMM regression model considered here was as follows (1):

1 $$\begin{aligned} & {\text{Transaminase}} \sim {\text{Sex}} + {\text{s}}\left( {{\text{Age}}} \right) + {\text{Antiviral}}\;{\text{Treatment}} + {\text{Exitus}} + {\text{s}}\left( {{\text{IM}}} \right)\;*\;{\text{Exitus}} \\ & \quad + {\text{s}}\left( {{\text{Time}}} \right)*{\text{Exitus}} + {\text{s}}\left( {{\text{ID}},\;{\text{bs}} = {\text{re}}} \right), \\ \end{aligned}$$

where “Transaminase” is AST, ALT, GGT, or ALP, and “IM” is CRP, D-dimer, IL-6, or lymphocytes. In the above equation, s(IM)*Exitus represents the association of “Exitus” according to the IM curve, i.e., a possible different non-linear effect of IM for deceased and living patients, and the same for s(Time)*Exitus. “ID” indicates the patient, and “s(ID, bs = “re”)” indicates the random intercepts by the patient. In the graphs, the smoothed effects are shown centered and with a 95% confidence interval.

After these first models, a second GAMM for each transaminase was fitted, including only the statistically significant covariates from the previous models. Statistical significance was established for p-values less than 0.05 and, in the case of smoothed continuous covariates, for those ranges of the variable in which the estimated 95% confidence intervals do not include zero. For the categorical covariates, as well as for the continuous variables whose smoothed effect was linear, we reported the corresponding estimated β coefficient. The effective degrees of freedom (edf) were also reported for all covariates. The edf number represents the parameters needed to estimate the effect of a covariate, i.e., edf indicates the complexity of the functional form of the effect. When the effect is linear, the edf is approximately 1.

The temporal correlation between transaminase levels and IM was estimated using the bivariate copula generalized additive models for location, scale, and shape^21^. Such a correlation is presented by means of Kendall’s τ. The model for the correlation of each transaminase-IM pair is determined by the following equation (2):

2 $${\text{Cor}}\left( {{\text{Transaminase}},\;{\text{IM}}} \right) \sim {\text{Sex}} + {\text{s}}\left( {{\text{Age}}} \right) + {\text{Antiviral}}\;{\text{Treatment}} + {\text{Exitus}} + {\text{s}}\left( {{\text{Time}}} \right)*{\text{Exitus,}}$$

in which, once again, “Transaminase” is AST, ALT, GGT, or ALP, and IM is CRP, D-dimer, IL-6, or lymphocytes. In the above equation, “s(Time)*Exitus” represents the association of “Exitus” according to the IM curve, i.e., a possible different non-linear effect of time for the deceased and living patients. All the Box–Cox-transformed covariates followed a Gaussian distribution, and the CRP and lymphocytes each followed a gamma distribution. In this type of model, a copula function is needed to model the correlation (see^21^ for more details). For this study, we selected the best copula according to Akaike’s Information Criterion and the Bayesian Information Criterion.

All the statistical analyses were performed using the free statistical software R^22^, version 4.1.0, using the MASS package for the Box–Cox transformation, the mgcv package for the GAMM regression, the GJRM package for the correlation analysis, and ggplot2 for all the plots presented in this paper.

## Results

### General description of mortality and associated factors

During the study period, 212 patients were admitted to the hospital with COVID-19. Table 1 shows the sample’s demographic and clinical characteristics. The median age was 68 years, and 56% were men. In our series, 37 (17.4%) patients died. The patients who died were significantly older, and there was a significantly higher percentage of patients with a history of ischemic heart disease, diabetes, and a lower oxygen saturation rate during the hospitalization. There were no differences between the deceased and those who survived with respect to the treatment undergone, past HBV or HCV infection, and statin treatment. There were no differences in body mass index, although those who died tended to have a higher index. Fifty three (53%), 56%, 42% and 19% of the patients presented elevated AST, ALT, GGT and ALP respectively. Of those patients: 12%,50% and 38%,presented elevated transaminase levels with a hepatocellular, cholestatic, and mixed pattern, respectively. The surviving patients who presented a hepatocyte pattern had a significant increase in ALT. The median AST and ALP values were significantly higher in the patients who died. There was a significant difference in IL-6, D-dimer, CRP, and lymphocyte values between the patients who died and those who survived (Table 1).

**Table 1:** Baseline characteristics of the study population, stratified by outcome.

		Total	Exitus	Alive	*P* value
Period under study		01/03/2020—19-06-2020			
Number of patients		68.40 [56.58, 77.18]	n = 212	n = 37	n = 175
Age (years)		120 (56.60%)	82.52 [73.35, 86.65]	65.48 [55.10. 74.57]	< 0.001
Male		190 (89.62%)	27 (72.97%)	93 (53.14%)	0.030
Lopinavir/Ritonavir and/or Hydroxychloroquine		78 (36.79%)	31 (83.78%)	159 (90.86%)	0.233
Statins		19 (7.55%)	15 (45.86%)	63 (36.00%)	0.708
Ischemic Heart Disease		48 (22.64%)	9 (24.32%)	10 (5.71%)	0.001
Diabetes		1 (0.56%)	17 (45.95%)	31 (17.71%)	< 0.001
HCV*		12 (6.63%)	0 (0.00%)	1 (0.64%)	1.000
HBV**		13 (6.13%)	1 (4.35%)	11 (6.96%)	1.000
Chronic Liver Disease			0 (0.00%)	13 (7.43%)	0.131
High liver enzymes	AST	112/212 (52.83%)	19/37 (51.35%)	93/175 (53.14%)	0.858
	ALT	119/212 (56.13%)	10/37 (27.03%)	109/175 (62.29%)	< 0.001
	GGT	90/212 (42.45%)	13/37 (35.14%)	77/175 (44.00%)	0.364
	ALP***	23/121 (19.01%)	7/24 (29.17%)	16/97 (16.49%)	0.160
	Hepatocellular pattern	15/121 (12.40%)	2/24 (8.33%)	13/97 (13.40%)	0.078
	Cholestatic pattern	60/121 (49.59%)	17/24 (70.83%)	43/97 (44.33%)	
	Mixed pattern	46/121 (38.02%)	5/24 (20.83%)	41/97 (42.27%)	
High bilirubin		48 (22.64%)	10 (27.03%)	38 (21.71%)	0.518
AST		30 [22, 45]	37 [24, 59]	29 [22, 42]	< 0.001
ALT		35 [22, 61]	31 [18, 64]	36 [23, 60]	0.007
GGT		45 [26, 86]	59 [29, 102]	44 [26, 80]	0.007
ALP		111 [79, 166]	152 [98, 219]	102 [70, 146]	< 0.001
Bilirubin		0.5 [0.4, 0.8]	0.5 [0.4, 0.8]	0.5 [0.4, 0.8]	0.615
CRP		4.16 [1.27, 9.24]	7.82 [3.30, 15.23]	3.64 [1.08, 8.54]	< 0.001
D-Dimer		764 [470, 1411]	1262 [690, 4616]	717 [465, 4335]	< 0.001
IL-6		7.47 [7.40, 19.23]	7.40 [7.34, 17.68]	7.49 [7.43, 19.40]	< 0.001
Lymphocytes		16.30 [8.50, 24.80]	6.90 [4.90, 10.95]	18.85 [11.20, 27.02]	< 0.001
BMI (kg/m^2^)****		30.12 [27.22, 34.05]	32.71 [30.03, 34.18]	29.73 [26.91, 33.34]	0.089
Oxygen saturation (%)		96 [96, 97]	94 [94, 97]	96 [95, 97]	< 0.001

Age, AST, ALT, GGT, ALP, Bilirubin, CRP, D-Dimer, IL-6, Lymphocytes, BMI, and oxygen saturation: Median (interquartile range). (*) The effective “n” was a total of 180: 23 exitus and 157 living; ** The effective “n” was a total of 181: 23 exitus and 158 living. (***) The effective “n” was a total of 121: 24 exitus and 97 living. (****) The effective “n” was a total of 93 patients.

**Figure 1:**
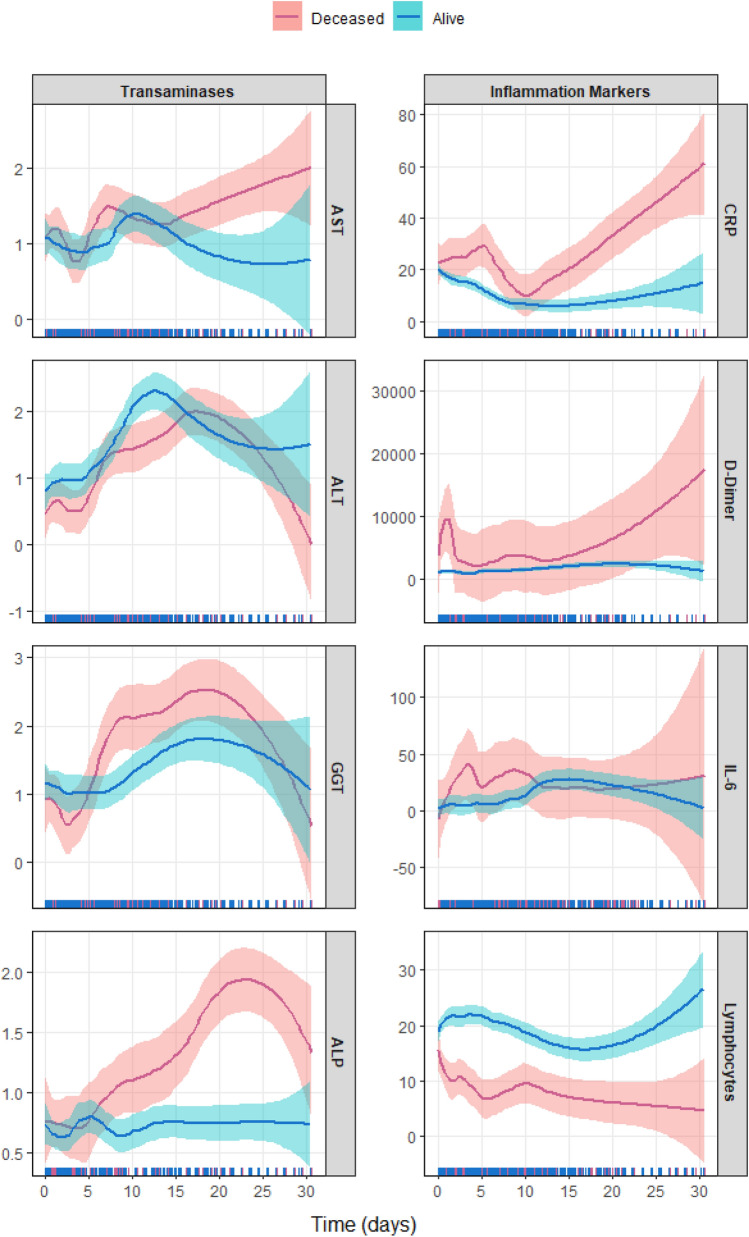
Exploratory curves for weighted values (when necessary) of transaminases (left) and inflammation markers (right) over time for deceased (pink) and living (blue) patients, along with 95% confidence intervals. Estimation made using a lasso regression analysis.

The temporal progression of enzyme and IM levels in the survivors and the deceased is shown in Fig. 1, which represents the mean estimate with a 95% confidence interval of these values over time and as a function of the outcome. The left part of the figure shows the transaminase behaviors in these patient groups. The patients who died showed a greater increase in transaminase levels than those who survived, although this increase was only notable for AST and ALP. Similarly, IMs showed a greater increase in the patients who died (right side of Fig. 1), although only CRP and D-dimer were significantly elevated compared with the levels in the patients who survived. Lymphocyte counts showed an inverse behavior, rising significantly in the patients who survived and decreasing significantly in the patients who died. This separation occurred throughout the disease’s progression.

The progression of transaminase levels at fixed intervals every 7 days during the first 3 weeks of hospitalization (Table 2) shows a characteristic elevation, from the first week, with respect to AST, ALT, GGT, and ALP, especially in those patients who died. The same progression for IMs also demonstrated a characteristic elevation for CRP and D-dimer, but from day zero, with a second elevation at the end of the observation period. This movement was less evident in the case of IL-6. Conversely, the lymphocytes count fell from the first week for the patients who died, with the differences being more intense between the second and third weeks compared with those of the survivors throughout the entire hospitalization period.

**Table 2:** Time course of transaminase levels according to final outcome.

		Day 0	Day 7	Day 14	Day 21
AST (UI/L)	Deceased	34 [20, 47]	69 [22, 89]	42 [39, 55]	40 [30, 50]
	Living	32 [25, 44]	28 [21, 44]	28 [24, 52]	24 [20, 29]
ALT (UI/L)	Deceased	20 [17, 33]	98 [28, 118]	71 [31, 150]	106 [90, 121]
	Living	33 [25, 52]	39 [27, 72]	66 [43, 137]	57[41, 74]
GGT (UI/L)	Deceased	39 [23, 70]	53 [46, 87]	110 [91, 125]	176 [138, 214]
	Living	42 [27, 64]	42 [27, 60]	74 [32, 102]	113 [83, 153]
ALP (UI/L)	Deceased	130[91, 265]	210 [181, 235]	111 [93, 197]	223[173, 272]
	Living	85 [63, 138]	124 [79, 157]	115 [80, 122]	88 [67, 94]
Bilirubin (mg/dL)	Deceased	0.6 [0.4, 0.8]	0.7 [0.5, 1.3]	0.4 [0.4, 0.8]	0.4 [0.4, 0.5]
	Living	0.5 [0.4, 0.7]	0.6 [0.4, 0.7]	0.4 [0.3, 0.5]	0.3 [0.3, 0.7]
CRP (mg/dL)	Deceased	7.8 [4.8, 12.1]	4.3 [1.3, 9.2]	9.2 [1.4, 18.2]	11.9 [6.0, 17.8]
	Living	6.0 [2.4, 10.2]	1.9 [0.8, 4.7]	1.3 [0.5, 3.5]	0.2 [0.1, 1.0]
D-dimer (ng/mL)	Deceased	949 [637, 1452]	835 [777, 1010]	897 [611, 1843]	3999 [3999, 3999]
	Living	644 [417, 1112]	709 [490, 1024]	1050 [582, 1596]	600 [361, 1096]
IL-6 (pg/mL)	Deceased	7.4 [7.4, 7.5]	7.5 [7.4, 76.8]	7.4 [7.3, 7.4]	7.3 [7.3, 7.4]
	Living	7.5 [7.4, 8.9]	7.5 [5.1, 10.1]	7.5 [7.5, 22.3]	7.4 [7.4, 44.5]
Lymphocytes (× 10^9^)	Deceased	11 [6, 18]	5 [2, 8]	5 [4, 5]	6 [5, 7]
	Living	18 [12, 27]	17 [8, 25]	18 [11, 21]	19 [11, 24]
O_2_ Sat (%)	Deceased	95 [93, 97]	94 [90, 97]	93 [91, 95]	–
	Living	96 [95, 98]	96 [95, 97]	96 [94, 97]	96 [96, 98]

Data are expressed in medians and interquartile ranges (between brackets).

### Multivariate analysis of transaminase values

In a preliminary analysis, each transaminase figure was evaluated individually with each of the IMs and adjusted for age, sex, and death, as well as for having undergone antiviral treatment. This analysis was repeated by performing a simultaneous adjustment between each of the transaminases and the 4 inflammation parameters considered, adjusting for the statistically significant variables in the first analysis. The results of this second analysis are shown in Table 3.

**Table 3:** Results for the multivariate regression models for the weighted and Box–Cox-transformed values of transaminases.

Outcome	Variables	Estimate (β)	SE	edf	P
AST (UI/L)	Intercept	− 2.697	1.090	1.000	0.014
	Exitus (Ref: Alive)	0.060	0.108	1.000	0.579
	D-dimer	0.952	0.452	1.000	0.036
	IL-6	0.102	0.029	1.000	< 0.001
	s(Age)	See Fig. 2		1.000	0.715
	s(Time) (Alive)	See Fig. 2		4.892	< 0.001
	s(Time) (Deceased)	See Fig. 2		1.001	0.270
ALT (UI/L)	Intercept	− 1.657	0.713	1.000	0.020
	Exitus (Ref: Alive)	− 0.251	0.126	1.000	0.046
	CRP	− 0.008	0.001	1.000	< 0.001
	D-dimer	0.579	0.265	1.000	0.029
	s(Age)	See Fig. 2		2.988	0.003
	s(Time) (Alive)	See Fig. 2		4.689	< 0.001
	s(Time) (Deceased)	See Fig. 2		3.965	< 0.001
GGT (UI/L)	Intercept	− 4.028	1.135	1.000	< 0.001
	Sex (Ref: Male)	0.339	0.126	1.000	0.008
	Exitus(Ref: Alive)	− 0.131	0.190	1.000	0.490
	CRP	− 0.002	0.002	1.000	0.373
	D-dimer	1.549	0.465	1.000	< 0.001
	IL-6	− 0.017	0.030	1.000	0.566
	Lymphocytes	− 0.007	0.003	1.000	0.021
	s(Age)	See Fig. 2		2.777	0.085
	s(Time) (Alive)	See Fig. 2		5.103	< 0.001
	s(Time) (Deceased)	See Fig. 2		4.194	< 0.001
ALP (UI/L)	Intercept	− 0.584	0.055	1.000	< 0.001
	Exitus (Ref: Alive)	0.242	0.131	1.000	0.065
	s(Age)	See Fig. 2		2.054	0.569
	s(Time) (Alive)	See Fig. 2		4.397	0.004
	s(Time) (Deceased)	See Fig. 2		3.871	< 0.001

SE means standard error. The edf number represents the parameters needed to estimate the effect of a covariate, indicating the complexity of the functional form of the smooth effect.

With regard to the time variable, ALT and ALP increased significantly, reaching a maximum from the second week in the patients who died. In addition, the AST levels decreased significantly and the GGT increased in those who survived. The latter drops significantly at the end of the observation period in both these patients who lived and in those who died.

**Figure 2:**
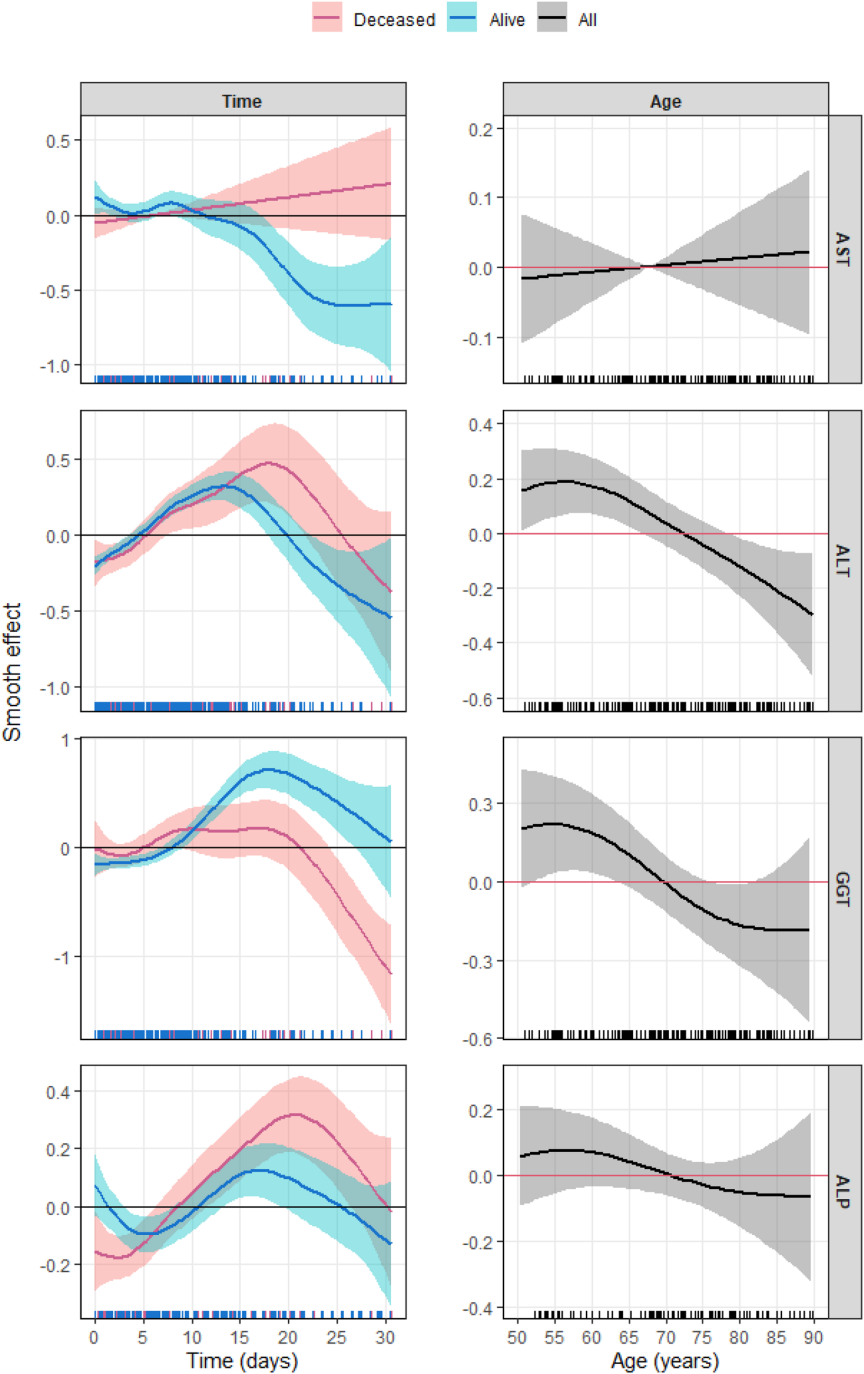
Estimated centered smooth effect (with 95% CI) of time (left) and age (right) over the weighted and Box–Cox-transformed transaminases extracted from the generalized additive multivariate models in Table 3. Pink stands for deceased patients, blue for the living, and black for the total number of patients. The horizontal line indicates zero, and when it lies inside the 95% CI, no statistical significance was found.

ALT is associated with age, increasing in middle age and decreasing significantly after the eighth decade. Despite its lack of statistical significance, the trend was similar in the analysis of age and the remaining transaminases. The following variables were statistically significant in this analysis: AST increased with IL-6 (*p* < 0.001), ALT decreased with CRP, and GGT decreased with lymphocytes. These 3 variables (AST, ALT and GGT) increased in turn with D-dimer (Fig. 2).

### Longitudinal correlations between transaminases and inflammation parameters

To assess the potential effect of time, drugs, sex, and age on the correlation between transaminases and IMs, we used the regression model expressed in equation^2^ (see “Statistical analysis” section). Table 4 shows the regression models, indicating only the statistically significant variables. Sex affected the correlation between D-dimer and AST and between D-dimer and ALT, increasing in women (β, 1.231 [*p* = 0.007] and β, 0.272 [*p* < 0.001], respectively). Sex also had an influence (but inverse), making the correlation lower in women between AST and lymphocytes, between ALT and CRP, between GGT and CRP, between GGT and lymphocytes, and between ALP and lymphocytes (β, − 0.450 [*p* = 0.020]; β, − 0.187 [*p* = 0.007]; β, − 0.234 [*p* = 0.023]; β, − 0454, [*p* = 0.028]; and β, − 2.118 [*p* = 0.010], respectively). Lastly, the patients who underwent antiviral therapy showed an increased correlation between D-dimer and GGT (β, 0.410; *p* = 0.046), and this same variable showed a decreased correlation between lymphocytes and ALP (β, − 2.118; *p* = 0.010). The association of these variables did not affect the final model.

**Table 4:** Regression models for correlation between weighted and Box–Cox-transformed values of transaminases and weighted and/or Box–Cox-transformed (if required) inflammation markers (†).

**Figure 3:**
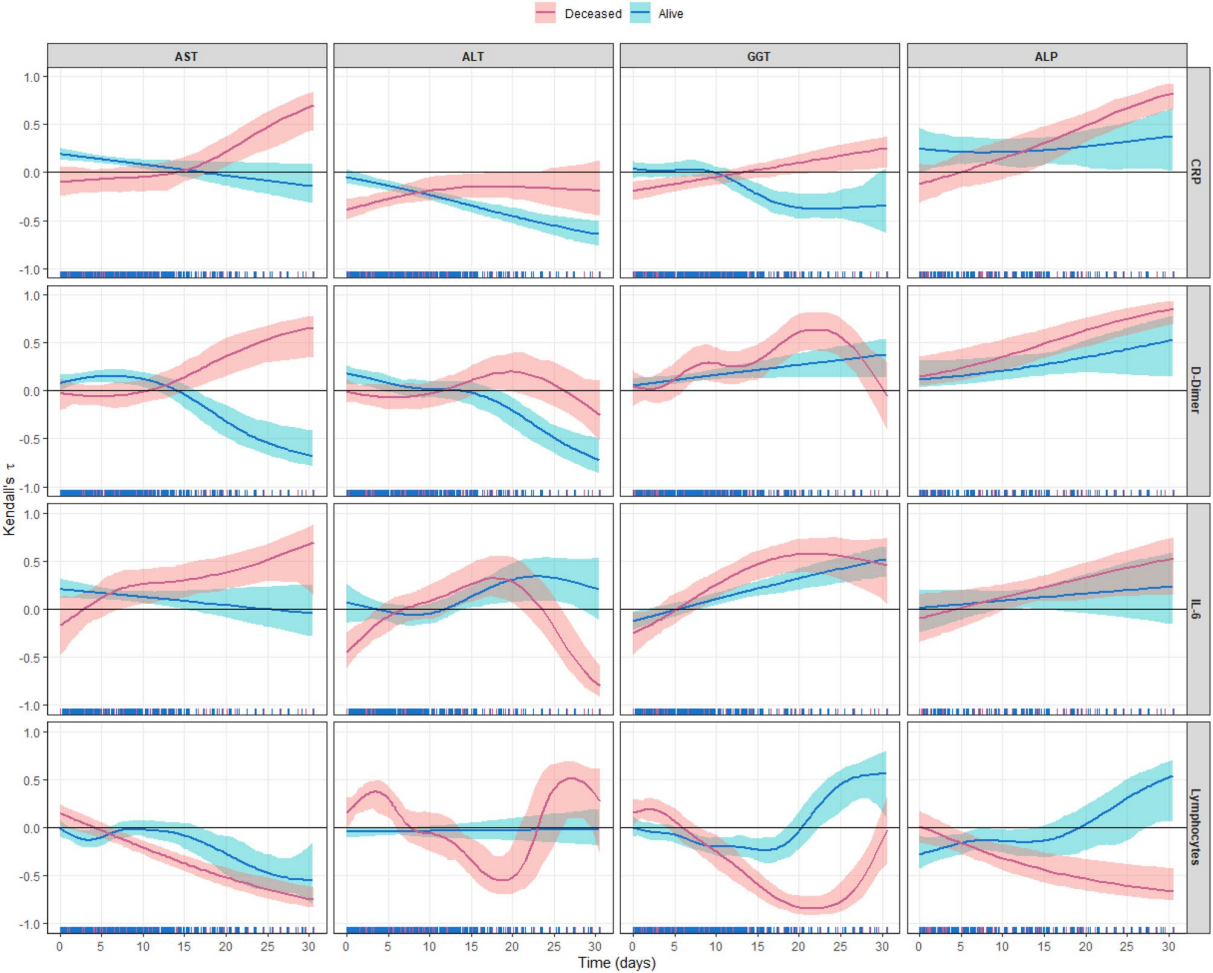
Time variation of the correlation between the weighted and Box–Cox-transformed transaminases and the weighted and/or Box–Cox-transformed (if required) inflammation markers for deceased (pink) and living (blue) patients, expressed by means of Kendall’s τ coefficient (with 95% CI). The horizontal line indicates zero correlation, and when it lies inside the 95% CI, no correlation was found.

**Figure 4:**
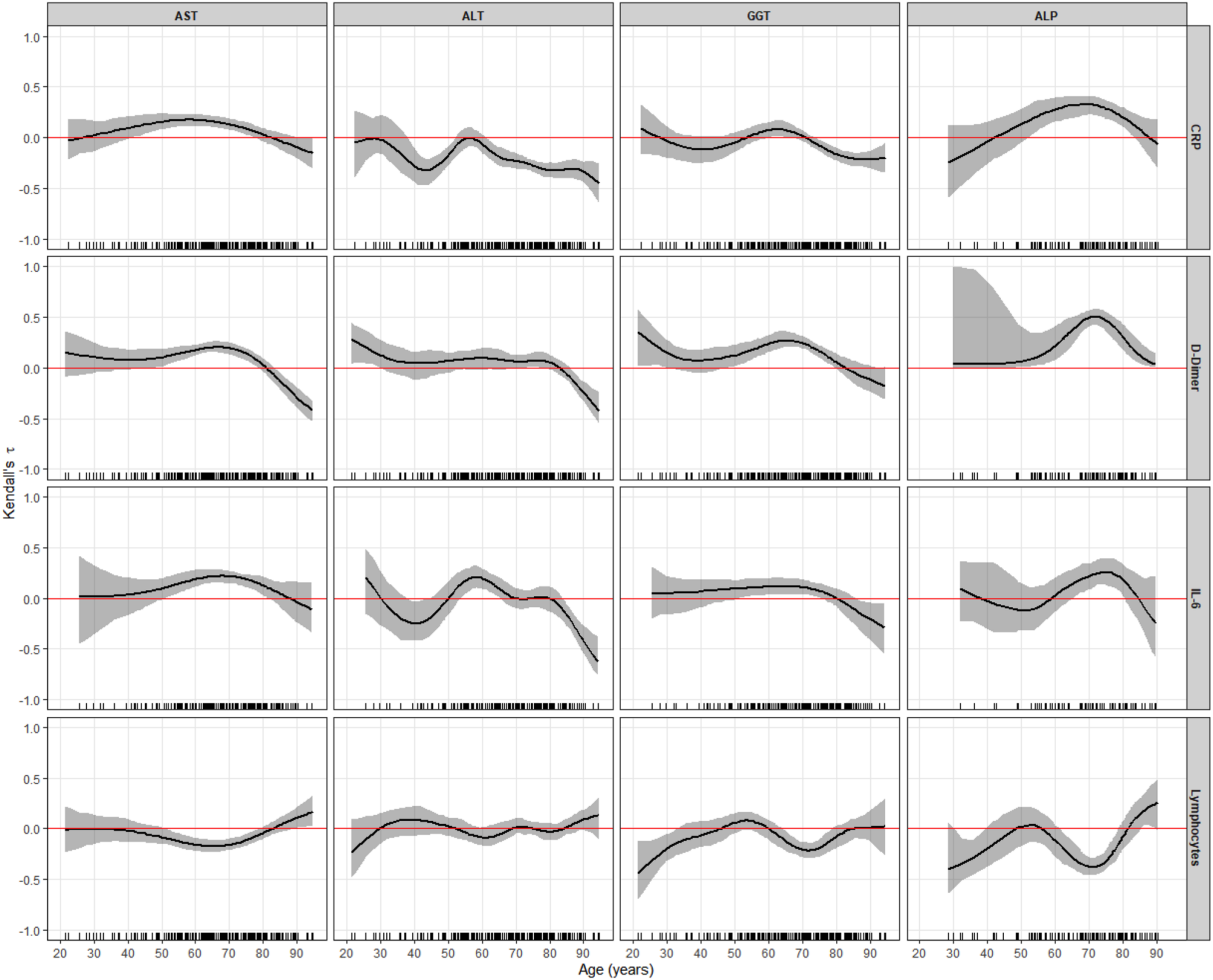
Variation of the correlation between the weighted and Box–Cox-transformed transaminases and the weighted and/or Box–Cox-transformed (if required) inflammation markers across the age of the patients, expressed by means of Kendall’s τ coefficient (with 95% CI). The horizontal red line indicates zero correlation, and when it lies inside the 95% CI, no correlation was found.

Cor(AST, CRP) ~ s(Age) + s(Time)*Exitus
Cor(AST, D-Dimer) ~ Sex + s(Age) + s(Time)* Exitus
Cor(AST, IL-6) ~ s(Age) + s(Time)*Exitus
Cor(AST, Lymphocytes) ~ Sex + s(Age) + s(Time)*Exitus
Cor(ALT, CRP) ~ Sex + s(Age) + s(Time)*Exitus
Cor(ALT, D-Dimer) ~ Sex + s(Age) + s(Time)* Exitus
Cor(ALT, IL-6) ~ s(Age) + s(Time)*Exitus
Cor(ALT, Lymphocytes) ~ Exitus + s(Time)*Exitus
Cor(GGT, CRP) ~ Sex + s(Age) + s(Time)*Exitus
Cor(GGT, D-Dimer) ~ Exitus + Antiviral Treatment + s(Age) + s(Time)* Exitus
Cor(GGT, IL-6) ~ s(Time)*Exitus
Cor(GGT, Lymphocytes) ~ Sex + s(Age) + s(Time)*Exitus
Cor(ALP, CRP) ~ Antiviral Treatment + s(Age) + s(Time)*Exitus
Cor(ALP, D-Dimer) ~ s(Age) + s(Time)* Exitus
Cor(ALP, IL-6) ~ s(Time)*Exitus
Cor(ALP, Lymphocytes) ~ Sex + s(Age) + s(Time)*Exitus

s() indicates the smooth effect of the covariate. * indicates association. For sex, the reference level is “Male”. For Exitus, the reference level is “Living”. For antiviral treatment, the reference level is “No”. The centered smooth effects of s(Time) and s(Age) in the correlation are depicted in Figs. 3 and 4, respectively. (†): From the general model type 2 every regression model in this table only includes the covariates that were found to be statistically significant (*p* < 0.05).

### Effect of time on the correlation between transaminases and inflammation parameters

The temporal correlation between transaminases and IMs (Fig. 3) showed a different dynamic between the survivors and the deceased. In the former (Fig. 3, in blue), CRP and AST showed a positive correlation that weakened from the second week of hospitalization. In this patient group and from the second week on, CRP and ALT showed a negative relationship, as did CRP and GGT. In contrast, the correlation between CRP and ALP in the surviving patients was positive. D-dimer showed an initially positive correlation to AST and ALT that became negative from the second week in the patients who survived. In contrast, the relationship between D-dimer, GGT, and ALP in the surviving patients was positive. IL-6 and AST in the surviving patients showed an initially positive correlation that weakened after 15 days. The correlation between IL-6 and GGT was also positive in these patients.

In the patients who died (Fig. 3, in pink), the correlation was positive between CRP and AST and between GGT and ALP, again after the second week. Although the correlation between D-dimer and ALT was not statistically significant, it was positive between AST, GGT, and ALP in the patients who died. In this group of patients, the correlation was significant and positive for IL-6 and AST, GGT, ALP, and ALT, although at the end of the observation period the correlation became negative for ALT.

Lastly, the lymphocyte counts in the survivors showed a negative correlation for AST and a positive one for GGT and ALP from the second week. In the patients who died, the correlation was statistically significant and negative for AST, GGT, and ALP and especially for ALT, although it became positive at the end of the observation period.

### Effect of age on the correlation between transaminases and inflammation parameters

Figure 4 shows the correlation between transaminases and IMs as a function of age; this correlation was lost in those older than 80 years. The AST and CRP correlation weakened in these older patients and became negative with ALT and GGT. With D-dimer and AST, ALT had a negative correlation in this age subgroup and again weakened in its relationship with GGT and FA, although the correlation with the latter continued to be positive. The correlation between IL-6, ALT, and GGT again became negative. In contrast to these markers in lymphocytes, the correlation expressed a positive trend as age advanced.

## Discussion

There is currently an abundance of articles indicating the high frequency of liver disorders in COVID-19. Nevertheless, it is still unclear why the liver is so frequently affected and what mechanism is ultimately involved^2–8,23–25^. Understanding why the liver experiences these changes is key to understanding the pathophysiology of COVID-19.

As with other studies, our study found that a high proportion (60%) of patients with COVID-19 had elevated transaminase levels. Unlike other studies, the percentage of patients with a hepatocellular and those with a cholestatic pattern was similar^3,25^. It is well documented that patients with previous liver damage appear more likely to present alterations with infection^5,11,24,26–29^. Our cohort was characterized by a low incidence of underlying liver disease, as well as of HCV and HBV infection. In addition, they were “first wave” patients treated with antivirals (lopinavir-ritonavir) and antimalarials (hydroxychloroquine) but not with immunosuppressive drugs such as corticosteroids. We believe that both characteristics facilitate the explanation of the potential mechanisms of damage, given that they are not masked by certain therapeutic interventions or previous pathology.

Another common finding in other studies is that older patients with ischemic heart disease and diabetes died more frequently from COVID-19. There is also a trend toward higher mortality in patients with higher body mass index. The missing data in this study have probably prevented us from observing a significant effect for obesity on prognosis, as reported in other publications^2,4,5,30,31^. We also observed that mortality was higher in those patients with greater higher increase in IMs, as has been reported in other studies^32,33^.

We found that the patients who died showed a significant increase in AST and ALP levels, whereas the patients who survived showed a significant decrease in AST levels. The increase in ALP levels and their temporal dynamics is especially striking. The increases in this enzyme in COVID-19 have been described in the medical literature as moderate^3,11^. The peculiar dynamics of ALP in this study have been revealed by the statistical methodology employed, given that we performed a continuous temporal analysis, the effects of which can be obviated in an analysis at a specific moment.

The mechanisms of liver damage have been associated with a direct action of the virus, the inflammatory response, hepatotoxicity, and anoxia^9–11^. Although the present study was not designed to determine the direct effects of the virus on its target organ, the statistical methodology applied allows us to infer the indirect mechanisms of damage. A correlation analysis found an association between IMs and transaminases. From the second week of hospitalization, the patients who died showed positive and significant 2-to-2 correlations between CRP and AST and between GGT and ALP. The D-dimer correlations are equally significant for this with AST, GGT, and ALP, which also occurs with IL-6. In contrast, the surviving patients showed negative correlations between CRP, ALT, and GGT, an effect that was equally significant in those patients who survived, from the second week of hospitalization, between D-dimer, AST, and ALT. The relationship between Il-6 and transaminases in these surviving patients is irrelevant.

In the patients who died, an inverse correlation between lymphocytes and AST, GGT, ALP, and even ALT is especially striking, although this effect was lost with this enzyme at the end of the observation period. Also of particular interest is the markedly negative correlation between ALP and lymphocytes for the patients who died and the positive correlation for those who survived. In adjusting the correlations with covariates, antiviral drugs exert a modifying effect, increasing the correlation between GGT and D-dimer and decreasing the correlation between ALT and CRP; however, these effects did not affect the form of the relationship. On the other hand, these drugs were administered in a similar proportion to the patients who lived and to those who died, which makes it impossible to attribute a significant effect to their use, both in terms of non-mortality and hepatotoxicity. We therefore believe that the treatment administered did not play a role in the liver damage. In this work, a sequential alteration of inflammatory markers followed by enzyme elevation has been detected, which could suggest that the latter is a consequence of inflammation, although a direct cytopathic effect cannot be ruled out.

This would justify the performance of specially directed studies to validate this hypothesis.

The observed cholestatic pattern of elevated ALP levels in the patients who died in our study is more biologically plausible given that the different distribution of specific receptors for the virus in hepatocytes and cholangiocytes is well documented and is predominant in the latter^10,11,24^.

The effect of age is particularly interesting. In a regression analysis, age behaved as a variable associated with ALT; ALT levels increase early in life and decrease from the eighth decade. When we established the correlations between age and the correlation itself with the markers of inflammation and transaminases, we observed that they became negative after the eighth decade. There is no clear biological explanation for this phenomenon, although the immunological senescence observed in vaccine trials could point to the effect^34,35^. Patients with higher increases in inflammation parameters showed higher mortality^32,33^, which contrasts with the fact that older patients despite showing less inflammation die more frequently; however, it is also true that older patients are affected by higher rates of diabetes, obesity, and heart disease, which influence mortality.

Various studies with longitudinal measurements of transaminases have been published, and a number of them have included inflammation parameters^12–16^. In a cross-sectional study, Phipps et al. found a relationship between ferritin and IL-6 with severe liver damage in a large cohort of patients with COVID-19^3^. Another longitudinal study established a relationship between ferritin and AST elevation but not with other IMs^12^. Another study observed a relationship between leukocyte counts, decreased lymphocyte counts, and increased transaminase levels^35^. In the remaining publications, specific associations were found between inflammation and liver disorders on one hand and mortality on the other but not specifically between IMs and liver disorders^14–16,27^.

In the present study, we used innovative flexible regression joint modeling techniques that, applied in this area, allow us to assess the dynamics of the temporal correlation between IMs and elevated transaminase levels. Furthermore, the advantage of this statistical methodology is that it allows us to adjust the temporal correlation for covariates such as age, sex, and treatment administration. For the successful application of this methodology, an exhaustive data capture was crucial, in which the measurements have been taken continuously over time, employing a specifically designed database. The use of these techniques has allowed us to establish a dynamic temporal representation of transaminases different from that of other studies. As far as we know, ours is the first study to use this type of tool to analyze liver disorders in SARS-CoV-2.

The retrospective nature of this study limits our conclusions. Despite exhaustive data capture, certain data was lacking to establish more robust conclusions. We also lacked histological studies. A histology compatible with immune damage would reinforce our conclusions. In addition, we only analyzed a certain IM battery, which limits the conclusions to the analyzed markers, although the statistical techniques employed can be extended to a larger panel of markers.

In conclusion, the present study once again confirms the frequent alteration of transaminase levels in patients with COVID-19 infection. A joint-modeling statistical methodology established that the patients who died from COVID-19 had characteristically elevated AST and ALP levels and showed a significant and sequential correlation over time with IMs. The patients who survived were better protected from inflammation-mediated liver damage. This relationship between transaminases and IMs weakened with age.

## Data Availability

The datasets used and/or analysed during the current study available from the corresponding author on reasonable request.
